# Supplementary material for: DNA repair is essential for Vibrio cholerae growth on thiosulfate–citrate–bile salts–sucrose (TCBS) medium
Source: J Bacteriol. 2025 Mar 26;207(4):e00004-25. doi: 10.1128/jb.00004-25 (PMC12004951; doi:10.1128/jb.00004-25)

Table S1: Mutations in *exoVII* suppressor mutants.

position	mutation	annotation	gene	NG001	supp 1	supp 2	supp 4	supp 5
280,469	(C) 5 → 6	pseudogene (639/1358 nt)	*glpT* →	[grey]	[grey]	[grey]	[grey]	[grey]
884,275	+GAG	coding (1700/2685 nt)	*gyrA* →				[green]	
885,040	T → G	I822S (ATC → AGC)	*gyrA* →					[green]
1,010,731	(T) 8 → 7	pseudogene (368/1948 nt)	*GPY04_RS04605* ←	[grey]	[grey]	[grey]	[grey]	[grey]
1,747,203	+GT	coding (1598/1941 nt)	*GPY04_RS07870* ←		[green]			
2,047,612	(C) 7 → 8	intergenic (-90/+155)	*GPY04_RS09340* ← / ← *dinB*	[grey]	[grey]	[grey]	[grey]	[grey]
2,221,809	(A) 9 → 8	intergenic (-60/+65)	*glnE* ← / ← *PY04_RS10050*	[grey]	[grey]	[grey]	[grey]	[grey]
2,510,903	+C	pseudogene (1737/2024 nt)	*gspD* ←	[grey]	[grey]	[grey]	[grey]	[grey]
2,567,382	T → C	K43R (AAA→AGA)	*rpsL* ←	[grey]	[grey]	[grey]	[grey]	[grey]
2,596,084	(T) 10 → 9	intergenic (-250/+6)	*hemE* ← / ← *nudC*	[grey]	[grey]	[grey]	[grey]	[grey]
2,830,015	C → T	P405S (CCC → TCC)	*gyrB* →				[green]	
2,830,195	Δ9 bp	coding (1018-1026/2418 nt)	*gyrB* →			[green]		

[grey] = found in NG001 parent strain

[green] = suppressor mutation

Figure S1.) Ox bile induces DNA damage in *V. cholerae*.

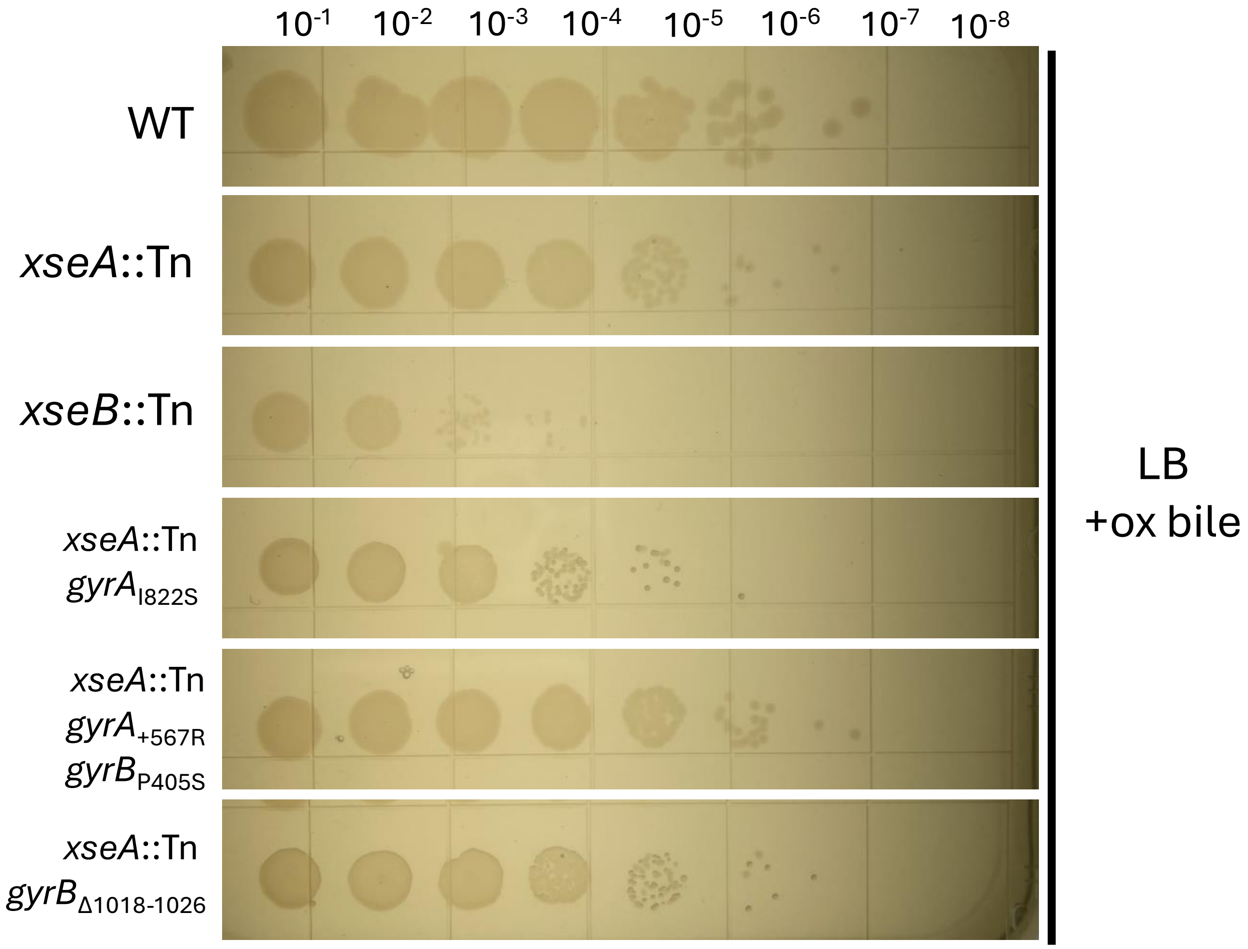

Figure S2.) *V. cholerae* mutants attenuated for growth in TCBS broth.

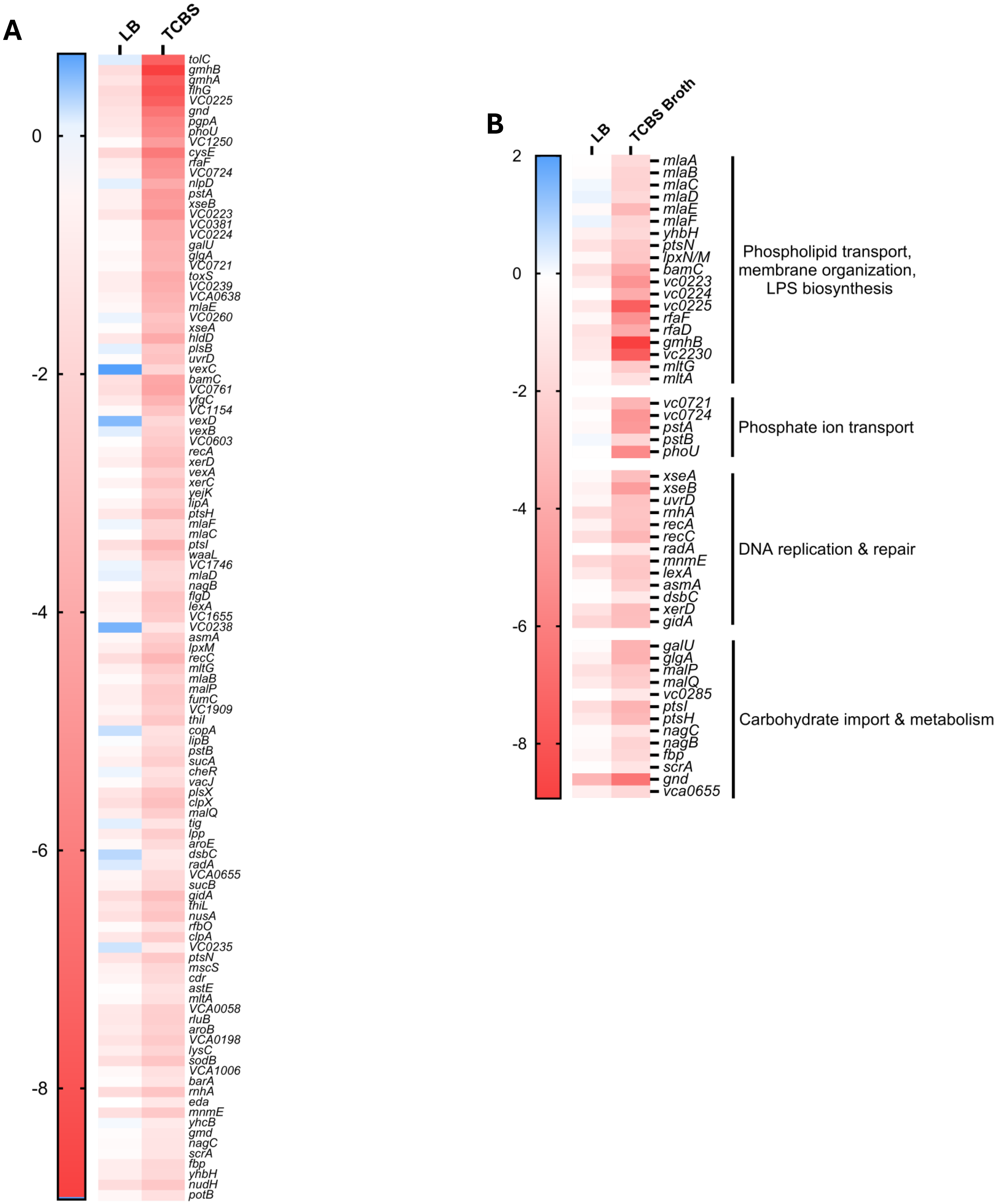

Supplement: Table S1; Figure S1 and S2 — Table S1: Mutations in exoVII suppressor mutants. Figure S1: Ox bile induces DNA damage in V. cholerae. Figure S2: V. cholerae mutants attenuated for growth in TCBS broth. [file jb.00004-25-s0001.pdf]
